# Modeling the effect of an associated single-nucleotide polymorphism in linkage studies

**DOI:** 10.1186/1471-2156-6-S1-S46

**Published:** 2005-12-30

**Authors:** Jeanine J Houwing-Duistermaat, Hae-Won Uh, Jeremie JP Lebrec, Hein Putter, Li Hsu

**Affiliations:** 1 Department of Medical Statistics and Bioinformatics, Leiden University Medical Center, Leiden, The Netherlands; 2 Modeling and Methods, Biostatistics Program, Fred Hutchinson Cancer Research Center, Seattle, Washington, USA

## Abstract

For linkage analysis in affected sibling pairs, we propose a regression model to incorporate information from a disease-associated single-nucleotide polymorphism located under the linkage peak. This model can be used to study if the associated single-nucleotide polymorphism marker partly explains the original linkage peak. Two sources of information are used for performing this task, namely the genotypes of the parents and the genotypes of the siblings. We applied the methods to three significantly disease-associated single-nucleotide polymorphisms and five microsatellite markers at the end of chromosome 3 of replicate 1 of Aipotu population. Two out of five of the microsatellite markers showed a LOD score higher than 3. The question to be answered was whether one of the single-nucleotide polymorphisms partly explains these high LOD scores. We did not have the answers when we analyzed the data.

## Background

When a region of interest is identified by a linkage study, one may proceed by typing single-nucleotide polymorphisms (SNPs) in the region and test whether these SNPs are associated with the outcome. If a SNP is significantly associated with the outcome, the question arises whether the identified SNP partly explains the original linkage peak. For quantitative traits observed in randomly selected siblings, Beekman et al. [1] proposed that a regression of each sibling pair's phenotype should be performed on their genotypes at the SNP locus and then a linkage analysis should be performed on the microsatellite markers using these residuals. If the SNP indeed explains the linkage peak, the original peak should become lower or even disappear. For a design consisting of only affected sibling pairs, this approach cannot be followed because of homogeneous phenotypes. However, one can study whether the linkage signal depends on the siblings' genotypes for the associated SNP.

Li et al. [2] proposed to assign a weight to each affected sibling pair according to their SNP genotypes and then test whether these weights are correlated with the scoring function *S*_*pairs *_at a microsatellite marker [3]. For an additive SNP effect, they proposed to use a weight proportional to the total number of risk alleles carried by the affected sibling pair. They showed that for sibling pairs this weight is uncorrelated to the number of alleles shared identically by descent (IBD). A strong correlation between these weights and *S*_*pairs *_[3] indicates the associated SNP may partly explain the linkage signals at the microsatellite markers.

Another source of information is the SNP genotypes of the parents. When both parents are homozygous for the SNP, the SNP genotypes of the affected siblings are fixed (nonrandom). In the extreme situation of one causal SNP or a SNP in complete linkage disequilibrium (LD) with the causal SNP, the IBD status at the microsatellite marker is not informative for transmission from parents homozygous for the SNP to affected offspring at the SNP locus. Hence the IBD probabilities of affected offspring of these parents are the probabilities under the null hypothesis. On the other hand, for affected siblings with heterozygous parents, the IBD status at the microsatellite marker is informative for transmission of the risk allele to the affected offspring. The risk allele will be most likely transmitted to the affected offspring and hence the allele at a linked microsatellite marker with the same grandparental origin will likely be transmitted. Based on this argument, Dupuis and Van Eerdewegh [4] proposed a test statistic to compare the linkage signal from offspring of homozygous parents with the linkage signal from offspring of heterozygous parents. When a significant difference exists, it can be concluded that the SNP partly explains the linkage peak.

As an alternative to the approaches of Dupuis and Van Eerdewegh [4] and Li et al. [2], in this report we propose the use of a regression model including a covariate that is based on the genotypes of the parents and of the siblings, respectively [5]. The advantage of using a regression model is that parameter estimates are obtained and that in these models other covariates (e.g., age, sex, and other known candidate genes) can easily be included. For example Holmans [6] showed that inclusion of known susceptibility genes may increase the power of linkage studies. We apply this approach to investigate whether the linkage signals at the end of chromosome 3 can be partly explained by one of the associated SNP.

## Methods

Olson [5] showed that the likelihood ratio (LR) of Risch [7] could be written as the likelihood ratio corresponding to a mixture of conditional-logistic models. For a sibling pair *j*,



with *α*_*i *_the prior probabilities that a sibling pair shares *i *alleles IBD and *f*_*ij *_the IBD status at the marker locus for sibling pair *j*. If the IBD status cannot be derived with certainty, *f*_*ij *_are the posterior weights. The parameter β_0 _is set to zero to avoid nonidentifiability. Depending on the underlying genetic model, constraints may be set on β_1 _and β_2_. Here, we use an additive model, i.e., . This parameterization corresponds to the following relationship for the IBD sharing in the affected sibling pair: *z*_0 _= 0.25, *z*_1 _= 0.5, and z_2 _= 0.5 - 0.25. By using the parameterization proposed by Olson [5], centralized covariates *x *can be easily be added to LR (1):



Under the additive model the IBD sharing in the affected sibling pair depends on the covariate *x*: *z*_0_(*x*) = 0.25, *z*_1 _= 0.5 and *z*_2_(*x*) = 0.5 - 0.25. Note that LR (2) is an overall test of linkage, i.e., it tests the null hypothesis of *β*_1_ = *δ *= 0 versus the alternative. The difference between LR (2) and LR (1) can be used to test the model with the covariate x versus the model without x, i.e., the null hypothesis of *δ *= 0.

To verify if an associated SNP explains partly the linkage peak, we can incorporate the indicator function as a covariate. The indicator function is defined as one if both parents are homozygous for the SNP and zero otherwise. Thus the centralized *x *is this indicator function minus its mean μ in the sample. Note that μ is the frequency of sibling pairs with parents homozygous for the SNP. If *δ *is zero the IBD sharing at the microsatellite is similar in offspring with homozygous parents to offspring with at least one heterozygous parent and the SNP does not explain the linkage peak at all. For *δ *< 0, the sharing of marker alleles IBD is higher in siblings with at least one parent heterozygous for the SNP compared with offspring of parents homozygous for the SNP. If *δ *is significantly smaller than zero, it can be concluded that the SNP partly explains the linkage peak.

If the genotypes of the parents are not available, the genotypes of the siblings can be used. We can study if the sharing of marker alleles IBD depends on the siblings' genotypes. We propose to use the centralized number of carried 'risk' alleles by the sibling pair as covariate *x*. Thus *x *is the number of risk alleles, which varies from 0 to 4, minus its mean in the sample of affected sib pairs. By doing so, we assume an additive model for the SNP [2], i.e., a multiplicative effect in the number of risk alleles on genetic relative risk. Again, if *δ *is zero the SNP does not explain the linkage peak at all. For *δ *> 0 the IBD sharing is higher in siblings who carry a high number of risk alleles and for *δ *< 0 the IBD sharing is higher in siblings who carry a low number of risk alleles. The models were fitted using the package SAGE 4.5 [8]. *P*-values smaller than 0.05 were considered to be statistically significant.

## Results

We applied the methods to the SNPs B03T3056, B03T3057, and B03T3066 and the five microsatellite markers at the end of chromosome 3 using replicate 1 of population Aipotu. This region was identified by performing linkage analysis of the micro-satellites using the affected sibling pairs from several replicates (see also Hsu et al. [9]). In replicate 1, a single point LOD score of 4.51 and of 3.06 were obtained for marker D3S0124 and marker D3S0127 respectively. Now Putter et al. [10] and Hsu et al. [9] identified three SNPs associated with the outcome in this replicate (B03T3056, B03T3057, and B03T3066) using an additive model. The associated variants are common and have allele frequencies of 0.64, 0.43, and 0.61 in controls. When adjusting for multiple testing, only SNP B03T3056 was significant [9].

To determine whether any of these SNPs partly explains the original linkage peak, we included the indicator function of both parental genotypes being homozygous as covariate in the model. The results are given in Table 1. The LOD scores were only slightly increased. For SNP B03T3056, which was most promising based on the association analysis, offspring of homozygous parents appeared to share more alleles IBD than offspring of heterozygous parents at all microsatellite markers. The estimate of the parameter *δ *at marker D3S0127 was  = 0.56 (standard error of 0.89).

Second, we considered the genotypes of the siblings as covariates. In line with the approach papers of Hsu et al. [9] and Putter et al. [10], an additive model was assumed for the SNP (see also [2]) and the covariate x was the centralized sum of the number of risk alleles carried by the sibling pair. The results are also given in Table 1. Adding B03T3056 to the model increased the LOD score significantly by 1.1 for marker D3S0127 (*P *= 0.02,  = 0.36). The LOD score at D3S0124 increased only by 0.64. Including the other SNPs in the model increased the LOD scores only slightly.

Finally we added the covariate based on the parental B03T3056 genotypes to the model in addition to sibling's B03T3056 genotypes. For the microsatellite marker D3S0127, the LOD score increased by 0.14. The corresponding estimate for the sibling's genotype was similar to the first estimate ( = 0.34) and the estimate for the parental genotype was smaller but still positive ( = 0.21).

## Discussion

In the original affected sibling pair linkage study, microsatellite markers D3S0124 and D3S0127 showed LOD scores above 3. In association analyses [9,10], SNP B03T3056 was highly significantly associated to the disease and B03T3057 and B03T3066 showed some significant association. In this paper, we modelled the IBD sharing at the two microsatellite markers and three neighboring microsatellite markers as a function of these SNP genotypes of the parents and of the sibling's genotypes. Only including the number of risk alleles of B03T3056 carried by the two siblings as covariate in the linkage analysis of marker D03S0127 increased the LOD score significantly (LOD score of 1.1, *P *= 0.02). From this analysis we conclude that only B03T3056 significantly explained a small part of the linkage signal. Other unknown genetic factors, probably in LD with B03T3056, are likely to be present in this region.

Including the parental B03T3056 genotypes in the linkage analysis of marker D3S0127 increased the LOD score only with 0.25. Siblings of parents homozygous for the SNP even showed higher IBD sharing than siblings with at least one heterozygous parent. This result is somewhat unexpected and not in line with the significant result when using the sibling's genotypes of this SNP as covariate in the linkage analysis of marker D3S0127. To disentangle association signals using SNP genotypes of the siblings and of the parents, extended modelling will likely be needed. More research in this area will be fruitful in understanding the phenomenon observed in this data analysis.

For situations in which the SNP genotypes of the parents are indeed significant, the question arises whether residual linkage exists, i.e., whether the SNP explains all genetic variation in this region. When the SNP is the only causal factor in the region or in complete LD with the causal factor, the IBD sharing for offspring of parents homozygous for the SNP should be similar to the probabilities under the null hypothesis of no linkage. A statistic could be formulated to test this null hypothesis. However, our analysis of parental genotypes does not support this hypothesis and therefore we did not perform such an analysis for residual linkage in these data.

This paper is a first attempt to combine the information available in genotypes of the parents, the siblings, and the IBD status at a microsatellite marker to better understand the role of a significantly disease-associated SNP. After knowing the answers, the conclusion that B03T3056 only partly explained the linkage peak and that other unknown factors are present in this region was correct. In this sense the proposed method appeared to work well. However, more research will be needed to study the statistical properties and assumptions of the method.

## Conclusion

We conclude that SNP B03T3056 only partly explains the original linkage peak. Other unknown genetic factors are probably present in this region. The models of Olson [5] can be used to study whether a SNP indeed explains the original linkage peak. More research is needed to better combine the various sources of information.

## Abbreviations

GAW: Genetic Analysis Workshop

IBD: Identity by descent

LD: Linkage disequilibrium

LR: Likelihood ratio

SNP: Single-nucleotide polymorphism

## Authors' contributions

JJH-D performed the analyses and wrote the manuscript. JJH-D, H-WU, JJPL carried out the preliminary linkage analyses. All authors participated in the development of the methods and interpretation of the results of the analysis. All authors read and approved the final manuscript.

## Figures and Tables

**Table 1:** Single-point LOD scores at five microsatellite markers at the end of chromosome 3.

Model and SNPs	Chromosomes									
	
	D3S0123		D3S0124		D3S0125		D3S0126		D3S0127	
Null model (linkage only)										
	l_1_^a^		l_1_		l_1_		l_1_		l_1_	
	1.65		4.51		0.41		1.97		3.06	
Including parental SNP genotypes										
	l_2 _– l_1_^c^	l_2_^b^	l_2 _– l_1_	l_2_	l_2 _– l_1_	l_2_	l_2 _– l_1_	l_2_	l_2 _– l_1_	l_2_
B03T3056	0.33	1.98	0.22	4.73	0.10	0.51	0.42	2.39	0.25	3.31
B03T3057	0.00	1.65	0.02	4.53	0.04	0.46	0.02	1.99	0.03	3.09
B03T3066	0.17	1.82	0.03	4.54	0.11	0.54	0.00	1.97	0.42	3.48
Including siblings' SNP genotypes										
	l_2 _– l_1_^c^	l_2_^b^	l_2 _– l_1_	l_2_	l_2 _– l_1_	l_2_	l_2 _– l_1_	l_2_	l_2 _– l_1_	l_2_
B03T3056	0.60	2.25	0.64	5.15	0.02	0.43	0.18	2.15	1.10	4.16
B03T3057	0.81	2.46	0.13	4.64	0.17	0.58	0.49	2.46	0.11	3.17
B03T3066	0.78	2.43	0.39	4.90	0.66	1.07	0.10	2.07	0.15	3.21

^a^l_1_is LOD score under linkage only (one degree of freedom)

^b^l_2_is LOD score for linkage including SNP genotypes (two degrees of freedom)

^c^l_2 _– l_1_is LOD score for comparing model with SNP genotypes to model without SNP genotypes (one degree of freedom)
